# The emergence of *E. coli* ST906 harboring the *bla*_NDM-21_ gene in a maternity and infant hospital in Jiangsu, China

**DOI:** 10.1128/spectrum.02927-24

**Published:** 2025-03-10

**Authors:** Yuehua Gao, Yaozhi Qi, Yue Yang, Yuhan Sun, Yujie Zhu, Guoping Zhao, Fei Xia, Biao Tang

**Affiliations:** 1 Key Laboratory of Systems Health Science of Zhejiang Province, School of Life Science, Hangzhou Institute for Advanced Study, University of Chinese Academy of Sciences, Hangzhou, China; 2 College of Food and Bioengineering, Shaanxi University of Science and Technology, Xi'an, Shaanxi, China; 3 Department of Laboratory, Lianyungang Maternal and Child Health Hospital, Lianyungang, Jiangsu, China; 4 Center for Supramolecular Chemistry & Catalysis and Department of Chemistry, College of Science, Shanghai University, Shanghai, China; University of Saskatchewan, Saskatoon, Saskatchewan, Canada

**Keywords:** *Escherichia coli*, antimicrobial resistance, *bla*_NDM-21_, conjugation transfer

## LETTER

Antimicrobial resistance (AMR) represents a significant global public health threat, with carbapenem-resistant *Enterobacteriaceae* (CRE) being especially problematic. The World Health Organization (WHO) has identified CRE as one of the critical categories of bacteria in urgent need of new treatments (1). Carbapenem antibiotics have become one of the most important antimicrobial agents for treating severe bacterial infections due to their stability against β-lactamases and low toxicity. Despite the ban on carbapenem use in veterinary medicine, *bla*_NDM_ has been reported in animal sources and the environment (2–4). This highlights the widespread nature of carbapenem-resistant bacteria across humans, animals, and the environment, challenging the “One Health” approach aimed at integrated health management across these domains.

To date, 70 subtypes of *bla*_NDM_ have been identified globally (https://www.ncbi.nlm.nih.gov/pathogens/refgene/#gene_family:blaNDM, August 2024), with NDM-1 and NDM-5 being the most common (3, 5, 6). Unlike other variants, *bla*_NDM-21_ has an amino acid substitution within the active site ring of the β chain (7). Discovered in 2016, *bla*_NDM-21_ was first isolated from *Escherichia coli* ST617 in a patient with a urinary tract infection in China (7). The IncX3 plasmid, known for its narrow host range and high stability, carries *bla*_NDM-21_ and plays a crucial role in its dissemination (8). This study reports the complete IncX3 plasmid sequence of the *bla*_NDM-21_-carrying *E. coli* ST906 strain EC23-1020 and confirms its conjugative transfer capability.

In 2023, a total of 24 *E. coli* isolates were collected from a children’s hospital in Jiangsu Province, China. One of these isolates, *E. coli* EC23-1020, was identified as resistant to meropenem. This strain, collected from the urine sample of a woman in Lianyungang City, showed resistance to several antibiotics as determined by the broth microdilution method and interpreted according to Clinical and Laboratory Standards Institute (CLSI) guidelines (2020: M100-S30). Specifically, EC23-1020 is resistant to a variety of β-lactam antibiotics, including ampicillin, amoxicillin-clavulanate, ceftiofur, ceftazidime, and meropenem, as well as the sulfonamide antibiotic sulfisoxazole. However, it was found to be sensitive to gentamicin, spectinomycin, tetracycline, and florfenicol (see Table S1 for details).

The genome DNA of strain EC23-1020 was extracted using a DNA extraction kit (GENEray, Shanghai). Genome sequencing was performed on the Illumina NovaSeq 6000 and Oxford Nanopore GridION platforms. The assembly was performed using Unicycler v0.5.0. Functional gene analysis, annotation, and classification were carried out using the RAST server (http://rast.nmpdr.org). The CGE server ResFinder 4.6.0 (http://genepi.food.dtu.dk/resfinder) was used to identify acquired resistance genes, while MLST 2.0 (https://cge.food.dtu.dk/services/MLST/) was used to predict sequence types. Plasmid types were predicted using PlasmidFinder 2.1 (https://cge.food.dtu.dk/services/PlasmidFinder/). Additionally, Easyfig 2.2.5 (with an e-value threshold of 0.001 and a homology threshold of 99%) and BRIG 0.95 (with a homology threshold of 70%) were used for comparative plasmid analysis. The assembly shows that the chromosome length of the plasmid pEC1020-NDM-21 is 4.77 Mb and two plasmids of 46.16 and 5.17 kb. MLST 2.0 analysis identified EC23-1020 as ST906, marking the first report of this sequence type. This contrasts with ST167, the most common NDM-positive *E. coli* type, followed by ST410. The first and currently the only reported *E. coli* strain carrying *bla*_NDM-21_ is ST617 (7), highlighting the genetic diversity among *E. coli* strains carrying *bla*_NDM-21_.

**Fig 1:**
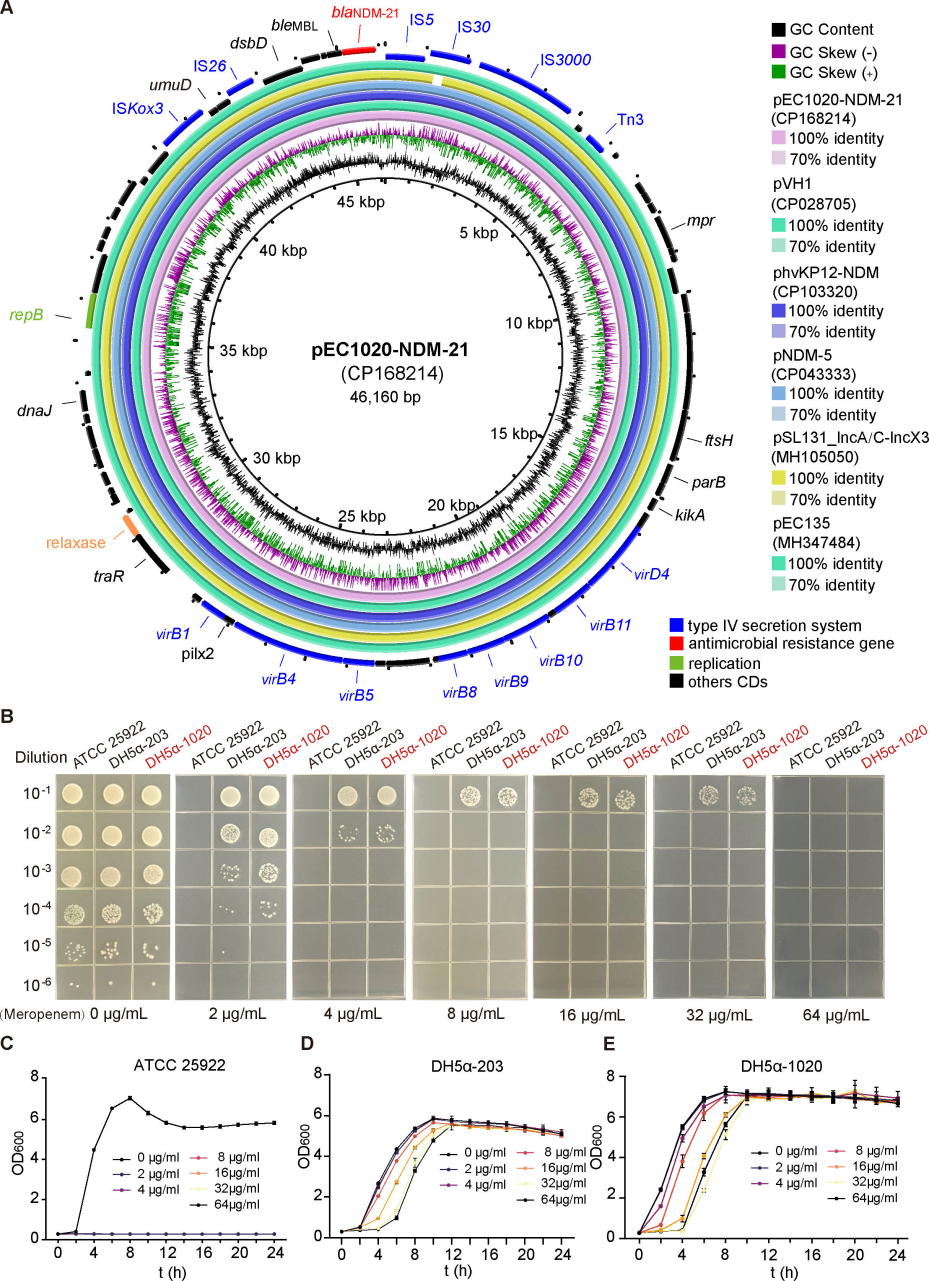
The sequence and phenotypic characteristics of plasmid pEC1020-NDM-21. (**A**). Comparative analysis of pEC1020-NDM-21 with similar plasmids. (**B**). Growth of strains ATCC 25922, DH5α−1020, and DH5α−203 on LB agar plates containing different concentrations of meropenem. Growth curves of strains ATCC 25922 (**C**), DH5α−203 (**D**), and DH5α−1020 (**E**) in LB containing different concentrations of meropenem.

The genome of the EC23-1020 isolate includes a circular chromosome and two circular plasmids. The *bla*_NDM-21_ gene resides on the IncX3 type plasmid pEC1020-NDM-21, which is 46.16 kb in length. *bla*_NDM-21_ is the sole acquired resistance gene in this isolate, embedded within the IS*30*-IS*5-bla*_NDM-21_-*ble*_MBL_-*trpF-dsbD*-IS*6* structure, as previously described (7). The plasmid features complete conjugative transfer elements, including the Type IV bacterial secretion system gene cluster (Fig. 1A). The plasmid pEC1020-NDM-21 exhibits 100% homology with plasmid pNDM21_020023 (CP025948), which harbors the *bla*_NDM-21_ gene and represents the first isolation of this plasmid type from *E. coli* in China (7). Additionally, plasmid pEC1020-NDM-21 demonstrates complete homology with the highly virulent *Klebsiella pneumoniae* plasmid phvKP12-NDM (CP103320) plasmid (9). The *bla*_NDM-21_ gene on the IncX3 plasmid, surrounded by mobile elements, mirrors that of *bla*_NDM_ found in other IncX3 plasmids (10). This high level of conservation suggests that *bla*_NDM-21_ may have evolved from *bla*_NDM-5_ through point mutations in this region.

In our previous study, we isolated plasmid pTB203 carrying *bla*_NDM-5_ from laying hens in Zhejiang Province (11). In this study, we used the TIANprep mini plasmid kit (TIANGEN, Beijing, China) extracted plasmids pTB203 and pEC1020-NDM-21 and transformed them into *E. coli* DH5α strains to generate transformants DH5α-203 and DH5α-1020 (12), which carry *bla*_NDM-5_ and *bla*_NDM-21_, respectively (Table 1). We assessed the growth of DH5α-203 and DH5α-1020 on LB agar plates with increasing concentrations of meropenem (0, 2, 4, 8, 16, 32, and 64 µg/mL). As depicted in Fig. 1B, both strains exhibited similar resistance patterns, with no growth observed at meropenem concentrations of 64 µg/mL. Growth curves (Fig. 1C through E) further confirmed that strain DH5α-1020 exhibited growth patterns consistent with plate assays, indicating no significant difference in meropenem resistance between the two strains. The *bla*_NDM-21_ gene in this study is located on an IncX3 plasmid, which, as reported previously, has a minimal adaptive cost to most hosts during inter-bacterial transmission (8), a finding corroborated by our results.

**TABLE 1:** Key bacterial strains used in this study

Strains	Description or relevant genotype
*E. coli* ATCC 25922	Control strain; laboratory storage
*E. coli* C600	Rifampicin-resistant; laboratory storage
*E. coli* EC23-1020	Constraining *bla*_NDM-21_-positive pEC1020-NDM-21; in this study
*E. coli* ECCRA-119	Previously isolated from layer faces; the isolate containing *bla*_NDM-5_-positive pTB203; laboratory storage
*E. coli* DH5α−1020	Strain DH5α harboring plasmid pEC1020-NDM-21; transformant in this study
*E. coli* DH5α−203	Strain DH5α harboring plasmid pTB203; transformant in this study

To evaluate conjugation, we used rifampin-resistant *E. coli* strain EC600 as the recipient strain, with a donor-to-recipient ratio of 1:1. Conjugation was performed on LB agar plates containing 400 µg/mL rifampin and 16 µg/mL meropenem for selection (13). Conjugants were identified by PCR and confirmed to contain the *bla*_NDM-21_ gene. The results indicated that *E. coli* EC600 could successfully receive meropenem resistance through conjugation, with an efficiency of (2.3 ± 0.32) × 10^−2^ (Fig. S1).

The continuous evolution of NDM enzymes may lead to the emergence of novel variants with differing hydrolytic activities against β-lactam antibiotics, exacerbating multidrug resistance in clinical pathogens. This escalating threat has significant implications for clinical medicine, veterinary practices, and food safety. Therefore, robust monitoring and control measures for bacteria harboring NDM genes are imperative.

## Data Availability

The complete genome sequences of strain EC23-1020 were deposited at GenBank under accession numbers CP168214-CP168216.

## SUPPLEMENTAL MATERIAL

Supplemental material spectrum.02927-24-s0001.docx Fig. S1; Table S1.

ASM does not own the copyrights to Supplemental Material that may be linked to, or accessed through, an article. The authors have granted ASM a non-exclusive, world-wide license to publish the Supplemental Material files. Please contact the corresponding author directly for reuse.
