# Supplementary material for: The emergence of E. coli ST906 harboring the blaNDM-21 gene in a maternity and infant hospital in Jiangsu, China
Source: Microbiol Spectr. 2025 Mar 10;13(4):e02927-24. doi: 10.1128/spectrum.02927-24 (PMC11960064; doi:10.1128/spectrum.02927-24)
Supplement: Supplemental material — Fig. S1; Table S1. [file spectrum.02927-24-s0001.docx]

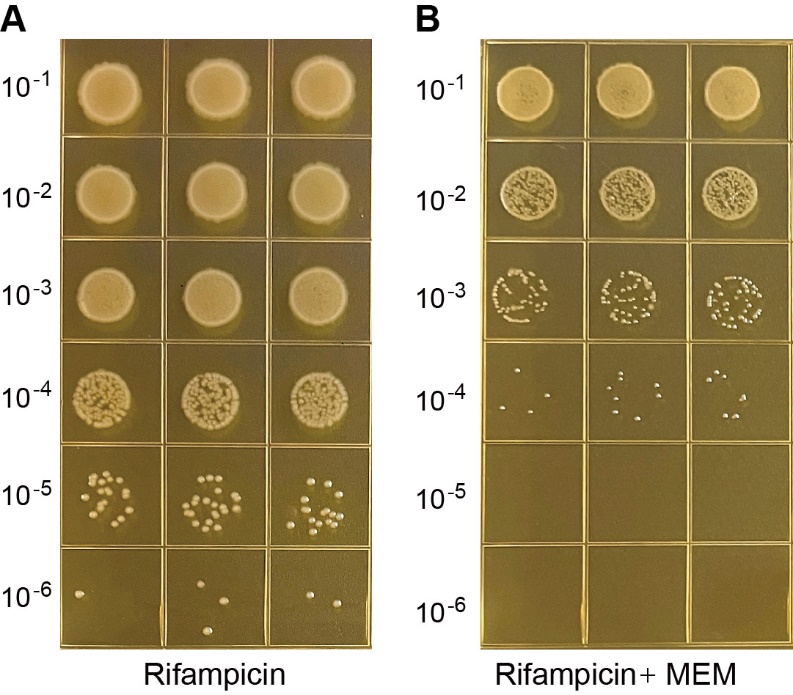


**Figure S1**. Conjugative transfer assay of strain EC23-1020. A and B represent the selection results under the selection pressure of rifampicin, rifampicin and meropenem, with strain C600, a rifampicin-resistant *E. coli* strain, as the recipient strain.

| **Antibiotic types** | **Antibiotics** | **MIC (mg/L)** | **Antimicrobial susceptibility** | **AMR gene** |
| --- | --- | --- | --- | --- |
| β-lactam | ampicillin (AMP) | ＞512 | resistant | *bla*_NDM-21_ |
|  | amoxicillin/clavulanate (A/C) | ＞512/256 | resistant | *bla*_NDM-21_ |
|  | ceftiofur (CEF) | ＞256 | resistant | *bla*_NDM-21_ |
|  | ceftazidime (CAZ) | ＞256 | resistant | *bla*_NDM-21_ |
|  | meropenem (MEM) | ＞16 | resistant | *bla*_NDM-21_ |
| aminoglycoside | gentamicin (GEN) | 1 | susceptible |  |
|  | Spectinomycin (SPT) | 32 | susceptible |  |
| fluoroquinolone | enrofloxacin (ENR) | 0.03 | susceptible |  |
|  | ofloxacin (OFL) | 0.06 | susceptible |  |
| tetracycline | tetracycline (TET) | 2 | susceptible |  |
| phenicol | florfenicol (FFC) | 4 | susceptible |  |
| sulfonamides | sulfisoxazole (SF) | ＞512 | resistant |  |
|  | trimethoprim/sulfamethoxazole (SXT) | ≤0.06/1.2 | susceptible |  |
| polymyxin | colistin (CS) | 0.25 | susceptible |  |

**Table S1**. The antimicrobial resistance of strain EC23-1020
